# Poly[(μ_2_-2,2′-bipyridine-κ^2^ *N*:*N*′)bis­(μ_3_-2,2,2-trifluoro­acetato-κ^3^ *O*:*O*:*O*′)disilver(I)]

**DOI:** 10.1107/S1600536810035282

**Published:** 2010-09-04

**Authors:** Hadi D. Arman, Tyler Miller, Edward R. T. Tiekink

**Affiliations:** a Department of Chemistry, The University of Texas at San Antonio, One UTSA Circle, San Antonio, Texas 78249-0698, USA; b Department of Chemistry, University of Malaya, 50603 Kuala Lumpur, Malaysia

## Abstract

In the title salt, [Ag_2_(CF_3_CO_2_)_2_(C_10_H_8_N_2_)]_*n*_, which may also be regarded as a coordination polymer if long Ag⋯O inter­actions are considered, each of the N atoms of the somewhat twisted 2,2′-bipyridine mol­ecule [N—C—C—N = −27.5 (4)°] binds to an Ag atom, and each of the carboxyl­ate ligands is tridentate, linking to three Ag atoms. The bidentate carboxyl­ate O atoms bridge the same two Ag atoms, resulting in the formation of Ag_2_O_2_ rings. These rings are bridged by the 2,2′-bipyridine ligands, forming a chain along the *b* axis. The chains are linked into double chains *via* the remaining Ag—O bonds and Ag⋯Ag contacts. As a consequence of the Ag⋯Ag contacts, the NO_4_ donor set about each Ag atom is heavily distorted. Finally, the chains are linked into a three-dimensional network by a combination of C—H⋯O and C—H⋯F inter­actions.

## Related literature

For structural diversity in the supra­molecular structures of silver salts, see: Kundu *et al.* (2010 ▶). For a related Ag salt, see: Arman *et al.* (2010 ▶).
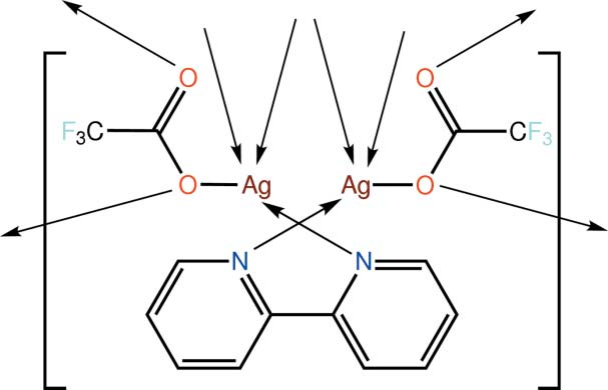


## Experimental

### 

#### Crystal data

[Ag_2_(C_2_F_3_O_2_)_2_(C_10_H_8_N_2_)]
*M* *_r_* = 597.96
Monoclinic, 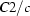

*a* = 24.597 (6) Å
*b* = 6.8474 (14) Å
*c* = 21.253 (5) Å
β = 116.029 (4)°
*V* = 3216.5 (13) Å^3^
*Z* = 8
Mo *K*α radiation
μ = 2.53 mm^−1^
*T* = 98 K
0.31 × 0.29 × 0.20 mm

#### Data collection

Rigaku AFC12/SATURN724 diffractometer
Absorption correction: multi-scan (*ABSCOR*; Higashi, 1995 ▶) *T*_min_ = 0.619, *T*_max_ = 1.000
10231 measured reflections
3662 independent reflections
3469 reflections with *I* > 2σ(*I*)
*R*_int_ = 0.037

#### Refinement

*R*[*F* ^2^ > 2σ(*F* ^2^)] = 0.031
*wR*(*F* ^2^) = 0.091
*S* = 1.09
3661 reflections
253 parameters
H-atom parameters constrained
Δρ_max_ = 0.66 e Å^−3^
Δρ_min_ = −0.65 e Å^−3^

### 

Data collection: *CrystalClear* (Molecular Structure Corporation & Rigaku, 2005 ▶); cell refinement: *CrystalClear*; data reduction: *CrystalClear*; program(s) used to solve structure: *SHELXS97* (Sheldrick, 2008 ▶); program(s) used to refine structure: *SHELXL97* (Sheldrick, 2008 ▶); molecular graphics: *ORTEP-3* (Farrugia, 1997 ▶) and *DIAMOND* (Brandenburg, 2006 ▶); software used to prepare material for publication: *publCIF* (Westrip, 2010 ▶).

## Supplementary Material

Crystal structure: contains datablocks global, I. DOI: 10.1107/S1600536810035282/hb5622sup1.cif

Structure factors: contains datablocks I. DOI: 10.1107/S1600536810035282/hb5622Isup2.hkl

Additional supplementary materials:  crystallographic information; 3D view; checkCIF report

## Figures and Tables

**Table 1:** Selected bond lengths (Å)

Ag1—O1	2.284 (2)
Ag1—N1	2.309 (3)
Ag1—O2^i^	2.323 (3)
Ag1—O3^ii^	2.844 (2)
Ag1—O2	2.993 (3)
Ag1⋯Ag1^i^	3.0675 (9)
Ag1⋯Ag2	3.1941 (7)
Ag2—O3	2.276 (2)
Ag2—O4^iii^	2.280 (3)
Ag2—N2	2.326 (3)
Ag2—O1^iv^	2.837 (2)
Ag2—O4	3.069 (3)
Ag2⋯Ag2^iii^	2.9687 (8)

Symmetry codes: (i) 
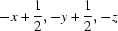
; (ii) 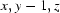; (iii) 
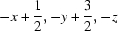
; (iv) 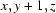.

**Table 2:** Hydrogen-bond geometry (Å, °)

*D*—H⋯*A*	*D*—H	H⋯*A*	*D*⋯*A*	*D*—H⋯*A*
C4—H4⋯O1^v^	0.95	2.50	3.410 (4)	159
C8—H8⋯O3^vi^	0.95	2.58	3.287 (4)	132
C1—H1⋯F6^ii^	0.95	2.54	3.072 (4)	116
C2—H2⋯F4^vii^	0.95	2.55	3.145 (4)	121
C10—H10⋯F3^iv^	0.95	2.52	3.076 (4)	117

Symmetry codes: (ii) 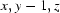; (iv) 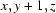; (v) 
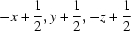
; (vi) 
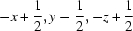
; (vii) 
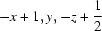
.

**Fig. 1.**
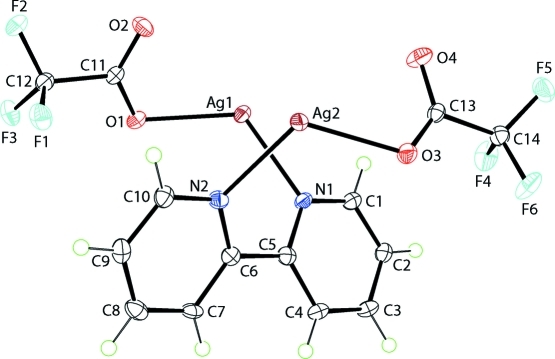
Asymmetric unit in the structure of (I) showing displacement ellipsoids at the 50% probability level.

**Fig. 2.**
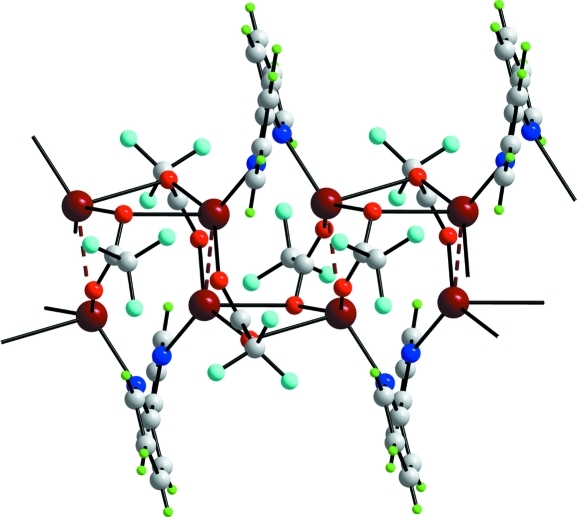
Portion of the supramolecular double chain aligned along the b axis in (I).

## supplementary crystallographic information

### Comment

Supramolecular structures of silver salts are highly dependent upon the nature of counter anions and the presence of solvent (Kundu *et al.*, 2010). During the course of on-going crystal engineering studies on silver salts (Arman *et al.*, 2010), the title salt was isolated and characterized.

The asymmetric unit of (I) comprises two Ag cations, a 2,2'-bipyridine molecule and two trifluoroacetate anions, Fig. 1. Each of the 2,2'-bipyridine-N atoms coordinates to a Ag atom bringing into close proximity the Ag1 and Ag2 atoms [Ag1···Ag2 = 3.1941 (7) Å]. In order to relive steric pressure, the 2,2'-bipyridine molecule is twisted as seen in the torsion angle [N1–C5–C6–N2 = -27.5 (4) °] and the dihedral angle formed between the pyridine rings of 26.93 (15) °. Each Ag atom forms a close Ag–O bond to a carboxylate-O, *i.e*. Ag1–O1 and Ag2–O3, Table 1, and each of the O1 and O3 atoms also bridges a neighbouring Ag atom to form a non-planar Ag_2_O_2_ ring. The second carboxylate-O atom in each case, *i.e.* O2 and O4, bridges to a different Ag atom so that each carboxylate ligand is tridentate. The supramolecular assembly is a double chain along the *b* axis whereby one row of Ag_2_O_2_ rings bridged by 2,2'-bipyridine molecules is connected to a second row *via* the Ag1–O2 and Ag2–O4 bonds and *vice versa*. Additional stability to the double chain is afforded by Ag1···Ag1 and Ag2···Ag2 interactions, Fig. 2 and Table 1. In terms of coordination geometry, each Ag atom exists within a NO_3_ donor set which is grossly distorted owing to the presence of two close Ag···Ag contacts. Chains are consolidated in the crystal packing by a combination of C–H···O and C–H···F contacts, Table 2.

### Experimental

2,2'-Bipyridine (0.015 g, 0.1 mmol) was dissolved in 5 ml of methanol. Added to this was silver trifluoroacetate (0.044 g, 0.2 mmol) dissolved in 7 ml of methanol. The resulting solution was gently heated and allowed to stand for slow evaporation affording colourless blocks of (I) after five days.

### Refinement

C-bound H-atoms were placed in calculated positions (C–H 0.95 Å) and were included in the refinement in the riding model approximation with *U*_iso_(H) set to 1.2*U*_eq_(C). In the final refinement a low angle reflection evidently effected by the beam stop were omitted, *i.e*. (200).

### Crystal data

**Table:**

[Ag_2_(C_2_F_3_O_2_)_2_(C_10_H_8_N_2_)]	*F*(000) = 2288
*M**_r_* = 597.96	*D*_x_ = 2.470 Mg m^−^^3^
Monoclinic, *C*2/*c*	Mo *K*α radiation, λ = 0.71069 Å
Hall symbol: -C 2yc	Cell parameters from 8570 reflections
*a* = 24.597 (6) Å	θ = 1.8–40.5°
*b* = 6.8474 (14) Å	µ = 2.53 mm^−^^1^
*c* = 21.253 (5) Å	*T* = 98 K
β = 116.029 (4)°	Block, colourless
*V* = 3216.5 (13) Å^3^	0.31 × 0.29 × 0.20 mm
*Z* = 8	

### Data collection

**Table:**

Rigaku AFC12K/SATURN724 diffractometer	3662 independent reflections
Radiation source: fine-focus sealed tube	3469 reflections with *I* > 2σ(*I*)
graphite	*R*_int_ = 0.037
ω scans	θ_max_ = 27.5°, θ_min_ = 1.8°
Absorption correction: multi-scan (*ABSCOR*; Higashi, 1995)	*h* = −25→31
*T*_min_ = 0.619, *T*_max_ = 1.000	*k* = −8→8
10231 measured reflections	*l* = −27→27

### Refinement

**Table:**

Refinement on *F*^2^	Primary atom site location: structure-invariant direct methods
Least-squares matrix: full	Secondary atom site location: difference Fourier map
*R*[*F*^2^ > 2σ(*F*^2^)] = 0.031	Hydrogen site location: inferred from neighbouring sites
*wR*(*F*^2^) = 0.091	H-atom parameters constrained
*S* = 1.09	*w* = 1/[σ^2^(*F*_o_^2^) + (0.0473*P*)^2^ + 8.2186*P*] where *P* = (*F*_o_^2^ + 2*F*_c_^2^)/3
3661 reflections	(Δ/σ)_max_ < 0.001
253 parameters	Δρ_max_ = 0.66 e Å^−^^3^
0 restraints	Δρ_min_ = −0.65 e Å^−^^3^

### Special details

**Table:**

Geometry. All s.u.'s (except the s.u. in the dihedral angle between two l.s. planes) are estimated using the full covariance matrix. The cell s.u.'s are taken into account individually in the estimation of s.u.'s in distances, angles and torsion angles; correlations between s.u.'s in cell parameters are only used when they are defined by crystal symmetry. An approximate (isotropic) treatment of cell s.u.'s is used for estimating s.u.'s involving l.s. planes.
Refinement. Refinement of *F*^2^ against ALL reflections. The weighted *R*-factor *wR* and goodness of fit *S* are based on *F*^2^, conventional *R*-factors *R* are based on *F*, with *F* set to zero for negative *F*^2^. The threshold expression of *F*^2^ > σ(*F*^2^) is used only for calculating *R*-factors(gt) *etc*. and is not relevant to the choice of reflections for refinement. *R*-factors based on *F*^2^ are statistically about twice as large as those based on *F*, and *R*- factors based on ALL data will be even larger.

### Fractional atomic coordinates and isotropic or equivalent isotropic displacement parameters (Å^2^)

**Table:**

	*x*	*y*	*z*	*U*_iso_*/*U*_eq_	
Ag1	0.270544 (11)	0.27690 (4)	0.078818 (12)	0.01708 (10)	
Ag2	0.239869 (12)	0.73275 (4)	0.063674 (12)	0.01661 (10)	
F1	0.05870 (11)	0.2464 (3)	0.01451 (13)	0.0250 (5)	
F2	0.04349 (10)	0.0721 (4)	−0.07648 (10)	0.0313 (5)	
F3	0.07622 (10)	−0.0612 (3)	0.02507 (11)	0.0243 (4)	
F4	0.45993 (11)	0.7568 (3)	0.16884 (13)	0.0287 (5)	
F5	0.45617 (9)	0.9732 (3)	0.09408 (11)	0.0298 (5)	
F6	0.43891 (9)	1.0540 (3)	0.18104 (11)	0.0296 (5)	
O1	0.18662 (10)	0.1008 (3)	0.06300 (11)	0.0186 (5)	
O2	0.15108 (12)	0.2233 (3)	−0.04584 (13)	0.0207 (5)	
O3	0.32741 (11)	0.9056 (3)	0.11732 (12)	0.0189 (5)	
O4	0.35046 (13)	0.7790 (4)	0.03384 (13)	0.0234 (5)	
N1	0.32454 (12)	0.4507 (4)	0.18047 (13)	0.0148 (5)	
N2	0.20388 (13)	0.5665 (4)	0.13321 (13)	0.0161 (5)	
C1	0.38483 (15)	0.4390 (5)	0.20200 (16)	0.0179 (6)	
H1	0.3991	0.4009	0.1689	0.021*	
C2	0.42676 (14)	0.4794 (5)	0.26943 (17)	0.0187 (6)	
H2	0.4688	0.4668	0.2826	0.022*	
C3	0.40639 (15)	0.5387 (5)	0.31752 (16)	0.0185 (6)	
H3	0.4343	0.5682	0.3643	0.022*	
C4	0.34421 (15)	0.5545 (4)	0.29617 (15)	0.0166 (6)	
H4	0.3291	0.5951	0.3283	0.020*	
C5	0.30455 (14)	0.5100 (4)	0.22711 (16)	0.0156 (6)	
C6	0.23773 (14)	0.5279 (4)	0.20229 (16)	0.0147 (6)	
C7	0.21148 (15)	0.5069 (4)	0.24806 (15)	0.0160 (6)	
H7	0.2361	0.4826	0.2964	0.019*	
C8	0.14863 (16)	0.5217 (5)	0.22253 (18)	0.0205 (6)	
H8	0.1300	0.5092	0.2531	0.025*	
C9	0.11430 (15)	0.5549 (5)	0.15166 (18)	0.0193 (6)	
H9	0.0715	0.5625	0.1325	0.023*	
C10	0.14338 (15)	0.5769 (4)	0.10896 (17)	0.0182 (6)	
H10	0.1195	0.6004	0.0604	0.022*	
C11	0.14613 (14)	0.1417 (4)	0.00329 (15)	0.0156 (6)	
C12	0.08041 (15)	0.0970 (5)	−0.00906 (16)	0.0174 (6)	
C13	0.36199 (14)	0.8644 (4)	0.09028 (15)	0.0149 (6)	
C14	0.42956 (15)	0.9154 (5)	0.13331 (16)	0.0174 (6)	

### Atomic displacement parameters (Å^2^)

**Table:**

	*U*^11^	*U*^22^	*U*^33^	*U*^12^	*U*^13^	*U*^23^
Ag1	0.01380 (15)	0.02195 (15)	0.01404 (15)	−0.00357 (9)	0.00477 (11)	−0.00392 (8)
Ag2	0.01414 (15)	0.02253 (15)	0.01274 (15)	−0.00263 (8)	0.00552 (11)	0.00048 (8)
F1	0.0216 (12)	0.0243 (10)	0.0344 (12)	0.0023 (8)	0.0171 (10)	−0.0020 (8)
F2	0.0195 (10)	0.0516 (14)	0.0162 (10)	−0.0099 (10)	0.0017 (8)	−0.0047 (9)
F3	0.0235 (10)	0.0207 (9)	0.0324 (11)	−0.0027 (8)	0.0158 (9)	0.0038 (8)
F4	0.0181 (11)	0.0223 (10)	0.0330 (13)	0.0050 (8)	−0.0005 (10)	0.0093 (8)
F5	0.0195 (10)	0.0442 (13)	0.0257 (11)	−0.0064 (10)	0.0100 (9)	0.0057 (9)
F6	0.0182 (10)	0.0323 (11)	0.0288 (11)	−0.0015 (8)	0.0017 (9)	−0.0153 (9)
O1	0.0154 (11)	0.0236 (11)	0.0126 (10)	−0.0014 (9)	0.0024 (9)	−0.0004 (8)
O2	0.0187 (13)	0.0276 (12)	0.0190 (12)	0.0018 (9)	0.0112 (11)	0.0058 (9)
O3	0.0144 (11)	0.0240 (11)	0.0180 (10)	−0.0016 (9)	0.0068 (9)	−0.0030 (9)
O4	0.0192 (13)	0.0307 (13)	0.0158 (12)	0.0028 (10)	0.0035 (11)	−0.0065 (9)
N1	0.0164 (12)	0.0141 (11)	0.0121 (11)	−0.0021 (10)	0.0045 (10)	−0.0019 (9)
N2	0.0171 (13)	0.0170 (12)	0.0133 (12)	0.0010 (10)	0.0058 (10)	0.0029 (9)
C1	0.0185 (15)	0.0191 (14)	0.0156 (14)	−0.0022 (12)	0.0070 (13)	−0.0038 (11)
C2	0.0140 (14)	0.0221 (15)	0.0185 (15)	−0.0027 (13)	0.0058 (12)	−0.0019 (12)
C3	0.0196 (16)	0.0204 (14)	0.0112 (13)	0.0010 (12)	0.0027 (12)	−0.0015 (11)
C4	0.0206 (16)	0.0149 (13)	0.0117 (13)	0.0010 (11)	0.0048 (12)	−0.0013 (11)
C5	0.0196 (15)	0.0117 (13)	0.0163 (14)	−0.0001 (12)	0.0086 (12)	0.0012 (10)
C6	0.0174 (14)	0.0102 (12)	0.0173 (14)	0.0011 (11)	0.0083 (12)	−0.0011 (10)
C7	0.0243 (16)	0.0141 (13)	0.0110 (13)	0.0007 (13)	0.0091 (12)	0.0022 (10)
C8	0.0291 (18)	0.0154 (14)	0.0245 (16)	−0.0005 (13)	0.0185 (15)	−0.0012 (12)
C9	0.0162 (15)	0.0160 (13)	0.0268 (16)	−0.0010 (12)	0.0105 (13)	−0.0021 (12)
C10	0.0175 (15)	0.0162 (14)	0.0185 (14)	−0.0010 (12)	0.0057 (13)	0.0018 (11)
C11	0.0137 (14)	0.0162 (13)	0.0138 (13)	−0.0009 (12)	0.0031 (11)	−0.0016 (11)
C12	0.0154 (15)	0.0203 (15)	0.0155 (14)	−0.0012 (12)	0.0059 (12)	−0.0027 (11)
C13	0.0119 (14)	0.0150 (13)	0.0142 (13)	0.0000 (11)	0.0024 (11)	0.0008 (11)
C14	0.0152 (15)	0.0184 (14)	0.0171 (14)	0.0006 (12)	0.0057 (12)	0.0008 (11)

### Geometric parameters (Å, °)

**Table:**

Ag1—O1	2.284 (2)	O4—Ag2^iii^	2.280 (3)
Ag1—N1	2.309 (3)	N1—C5	1.348 (4)
Ag1—O2^i^	2.323 (3)	N1—C1	1.349 (4)
Ag1—O3^ii^	2.844 (2)	N2—C10	1.346 (4)
Ag1—O2	2.993 (3)	N2—C6	1.359 (4)
Ag1—Ag1^i^	3.0675 (9)	C1—C2	1.377 (4)
Ag1—Ag2	3.1941 (7)	C1—H1	0.9500
Ag2—O3	2.276 (2)	C2—C3	1.382 (4)
Ag2—O4^iii^	2.280 (3)	C2—H2	0.9500
Ag2—N2	2.326 (3)	C3—C4	1.396 (5)
Ag2—O1^iv^	2.837 (2)	C3—H3	0.9500
Ag2—O4	3.069 (3)	C4—C5	1.394 (4)
Ag2—Ag2^iii^	2.9687 (8)	C4—H4	0.9500
F1—C12	1.348 (4)	C5—C6	1.495 (4)
F2—C12	1.328 (4)	C6—C7	1.391 (4)
F3—C12	1.333 (4)	C7—C8	1.400 (5)
F4—C14	1.346 (4)	C7—H7	0.9500
F5—C14	1.326 (4)	C8—C9	1.385 (5)
F6—C14	1.333 (4)	C8—H8	0.9500
O1—C11	1.254 (4)	C9—C10	1.388 (4)
O2—C11	1.237 (4)	C9—H9	0.9500
O2—Ag1^i^	2.322 (3)	C10—H10	0.9500
O3—C13	1.250 (4)	C11—C12	1.550 (4)
O4—C13	1.250 (4)	C13—C14	1.545 (4)
			
O1—Ag1—N1	121.42 (9)	C5—C4—C3	119.1 (3)
O1—Ag1—O2^i^	140.48 (9)	C5—C4—H4	120.5
N1—Ag1—O2^i^	94.01 (9)	C3—C4—H4	120.5
O1—Ag1—Ag1^i^	86.13 (5)	N1—C5—C4	121.9 (3)
N1—Ag1—Ag1^i^	149.60 (7)	N1—C5—C6	117.7 (3)
O2^i^—Ag1—Ag1^i^	65.77 (7)	C4—C5—C6	120.4 (3)
O1—Ag1—Ag2	110.04 (6)	N2—C6—C7	121.6 (3)
N1—Ag1—Ag2	66.67 (7)	N2—C6—C5	117.0 (3)
O2^i^—Ag1—Ag2	99.26 (6)	C7—C6—C5	121.4 (3)
Ag1^i^—Ag1—Ag2	93.284 (12)	C6—C7—C8	119.6 (3)
O3—Ag2—O4^iii^	143.51 (9)	C6—C7—H7	120.2
O3—Ag2—N2	118.43 (9)	C8—C7—H7	120.2
O4^iii^—Ag2—N2	93.99 (10)	C9—C8—C7	118.4 (3)
O3—Ag2—Ag2^iii^	85.12 (6)	C9—C8—H8	120.8
O4^iii^—Ag2—Ag2^iii^	70.16 (7)	C7—C8—H8	120.8
N2—Ag2—Ag2^iii^	151.77 (7)	C8—C9—C10	119.1 (3)
O3—Ag2—Ag1	109.14 (6)	C8—C9—H9	120.5
O4^iii^—Ag2—Ag1	98.63 (6)	C10—C9—H9	120.5
N2—Ag2—Ag1	66.22 (7)	N2—C10—C9	123.0 (3)
Ag2^iii^—Ag2—Ag1	92.459 (12)	N2—C10—H10	118.5
C11—O1—Ag1	107.24 (19)	C9—C10—H10	118.5
C11—O2—Ag1^i^	131.1 (2)	O2—C11—O1	128.8 (3)
C13—O3—Ag2	109.72 (19)	O2—C11—C12	115.3 (3)
C13—O4—Ag2^iii^	127.5 (2)	O1—C11—C12	115.7 (3)
C5—N1—C1	118.0 (3)	F2—C12—F3	107.7 (3)
C5—N1—Ag1	126.7 (2)	F2—C12—F1	107.7 (3)
C1—N1—Ag1	112.5 (2)	F3—C12—F1	106.1 (2)
C10—N2—C6	118.3 (3)	F2—C12—C11	112.1 (2)
C10—N2—Ag2	113.3 (2)	F3—C12—C11	113.1 (3)
C6—N2—Ag2	123.9 (2)	F1—C12—C11	109.9 (3)
N1—C1—C2	123.4 (3)	O4—C13—O3	129.3 (3)
N1—C1—H1	118.3	O4—C13—C14	114.0 (3)
C2—C1—H1	118.3	O3—C13—C14	116.7 (3)
C1—C2—C3	118.7 (3)	F5—C14—F6	107.3 (3)
C1—C2—H2	120.7	F5—C14—F4	106.7 (3)
C3—C2—H2	120.7	F6—C14—F4	106.2 (3)
C2—C3—C4	118.9 (3)	F5—C14—C13	113.3 (3)
C2—C3—H3	120.5	F6—C14—C13	113.1 (3)
C4—C3—H3	120.5	F4—C14—C13	109.7 (3)
			
O1—Ag1—Ag2—O3	159.39 (8)	C1—C2—C3—C4	0.3 (5)
N1—Ag1—Ag2—O3	42.84 (9)	C2—C3—C4—C5	0.1 (5)
O2^i^—Ag1—Ag2—O3	−47.55 (9)	C1—N1—C5—C4	−1.4 (4)
Ag1^i^—Ag1—Ag2—O3	−113.53 (6)	Ag1—N1—C5—C4	158.2 (2)
O1—Ag1—Ag2—O4^iii^	−44.62 (9)	C1—N1—C5—C6	178.3 (3)
N1—Ag1—Ag2—O4^iii^	−161.18 (10)	Ag1—N1—C5—C6	−22.1 (4)
O2^i^—Ag1—Ag2—O4^iii^	108.43 (9)	C3—C4—C5—N1	0.5 (5)
Ag1^i^—Ag1—Ag2—O4^iii^	42.45 (7)	C3—C4—C5—C6	−179.2 (3)
O1—Ag1—Ag2—N2	45.95 (9)	C10—N2—C6—C7	−2.5 (4)
N1—Ag1—Ag2—N2	−70.61 (11)	Ag2—N2—C6—C7	152.2 (2)
O2^i^—Ag1—Ag2—N2	−161.00 (10)	C10—N2—C6—C5	177.8 (3)
Ag1^i^—Ag1—Ag2—N2	133.03 (8)	Ag2—N2—C6—C5	−27.5 (4)
O1—Ag1—Ag2—Ag2^iii^	−114.92 (6)	N1—C5—C6—N2	−27.5 (4)
N1—Ag1—Ag2—Ag2^iii^	128.52 (7)	C4—C5—C6—N2	152.2 (3)
O2^i^—Ag1—Ag2—Ag2^iii^	38.13 (7)	N1—C5—C6—C7	152.8 (3)
Ag1^i^—Ag1—Ag2—Ag2^iii^	−27.845 (17)	C4—C5—C6—C7	−27.5 (4)
N1—Ag1—O1—C11	132.75 (19)	N2—C6—C7—C8	1.3 (4)
O2^i^—Ag1—O1—C11	−76.9 (2)	C5—C6—C7—C8	−179.0 (3)
Ag1^i^—Ag1—O1—C11	−33.61 (19)	C6—C7—C8—C9	0.7 (5)
Ag2—Ag1—O1—C11	58.5 (2)	C7—C8—C9—C10	−1.5 (5)
O4^iii^—Ag2—O3—C13	−78.8 (2)	C6—N2—C10—C9	1.7 (5)
N2—Ag2—O3—C13	131.3 (2)	Ag2—N2—C10—C9	−155.6 (3)
Ag2^iii^—Ag2—O3—C13	−32.30 (19)	C8—C9—C10—N2	0.3 (5)
Ag1—Ag2—O3—C13	58.6 (2)	Ag1^i^—O2—C11—O1	31.3 (5)
O1—Ag1—N1—C5	−20.2 (3)	Ag1^i^—O2—C11—C12	−153.5 (2)
O2^i^—Ag1—N1—C5	178.2 (2)	Ag1—O1—C11—O2	15.3 (4)
Ag1^i^—Ag1—N1—C5	132.1 (2)	Ag1—O1—C11—C12	−159.9 (2)
Ag2—Ag1—N1—C5	79.9 (2)	O2—C11—C12—F2	27.9 (4)
O1—Ag1—N1—C1	140.4 (2)	O1—C11—C12—F2	−156.2 (3)
O2^i^—Ag1—N1—C1	−21.3 (2)	O2—C11—C12—F3	149.9 (3)
Ag1^i^—Ag1—N1—C1	−67.4 (3)	O1—C11—C12—F3	−34.2 (4)
Ag2—Ag1—N1—C1	−119.6 (2)	O2—C11—C12—F1	−91.8 (3)
O3—Ag2—N2—C10	140.1 (2)	O1—C11—C12—F1	84.1 (3)
O4^iii^—Ag2—N2—C10	−22.5 (2)	Ag2^iii^—O4—C13—O3	26.8 (5)
Ag2^iii^—Ag2—N2—C10	−76.4 (3)	Ag2^iii^—O4—C13—C14	−156.6 (2)
Ag1—Ag2—N2—C10	−120.2 (2)	Ag2—O3—C13—O4	15.0 (4)
O3—Ag2—N2—C6	−15.7 (3)	Ag2—O3—C13—C14	−161.6 (2)
O4^iii^—Ag2—N2—C6	−178.3 (2)	O4—C13—C14—F5	38.5 (4)
Ag2^iii^—Ag2—N2—C6	127.8 (2)	O3—C13—C14—F5	−144.4 (3)
Ag1—Ag2—N2—C6	84.0 (2)	O4—C13—C14—F6	160.9 (3)
C5—N1—C1—C2	1.8 (5)	O3—C13—C14—F6	−22.0 (4)
Ag1—N1—C1—C2	−160.6 (3)	O4—C13—C14—F4	−80.7 (3)
N1—C1—C2—C3	−1.3 (5)	O3—C13—C14—F4	96.5 (3)

Symmetry codes: (i) −*x*+1/2, −*y*+1/2, −*z*; (ii) *x*, *y*−1, *z*; (iii) −*x*+1/2, −*y*+3/2, −*z*; (iv) *x*, *y*+1, *z*.

### Hydrogen-bond geometry (Å, °)

**Table:**

*D*—H···*A*	*D*—H	H···*A*	*D*···*A*	*D*—H···*A*
C4—H4···O1^v^	0.95	2.50	3.410 (4)	159
C8—H8···O3^vi^	0.95	2.58	3.287 (4)	132
C1—H1···F6^ii^	0.95	2.54	3.072 (4)	116
C2—H2···F4^vii^	0.95	2.55	3.145 (4)	121
C10—H10···F3^iv^	0.95	2.52	3.076 (4)	117

Symmetry codes: (v) −*x*+1/2, *y*+1/2, −*z*+1/2; (vi) −*x*+1/2, *y*−1/2, −*z*+1/2; (ii) *x*, *y*−1, *z*; (vii) −*x*+1, *y*, −*z*+1/2; (iv) *x*, *y*+1, *z*.

## References

[bb1] Arman, H. D., Miller, T., Poplaukhin, P. & Tiekink, E. R. T. (2010). *Acta Cryst.* E**66**, m1167–m1168. 10.1107/S1600536810033611 PMC3008117 21588558

[bb2] Brandenburg, K. (2006). *DIAMOND* Crystal Impact GbR, Bonn, Germany.

[bb3] Farrugia, L. J. (1997). *J. Appl. Cryst.* **30**, 565.

[bb4] Higashi, T. (1995). *ABSCOR* Rigaku Corporation, Tokyo, Japan.

[bb5] Kundu, N., Audhya, A., Towsif Abtab, Sk. Md., Ghosh, S., Tiekink, E. R. T. & Chaudhury, M. (2010). *Cryst. Growth Des.* **10**, 1269–1282.

[bb6] Molecular Structure Corporation & Rigaku (2005). *CrystalClear* MSC, The Woodlands, Texas, USA, and Rigaku Corporation, Tokyo, Japan.

[bb7] Sheldrick, G. M. (2008). *Acta Cryst.* A**64**, 112–122. 10.1107/S0108767307043930 18156677

[bb8] Westrip, S. P. (2010). *J. Appl. Cryst.* **43**, 920–925.

